# Recurrent Aseptic Meningitis From Herpes Simplex Virus-2: Mollaret's Meningitis in a 30-Year-Old Female

**DOI:** 10.7759/cureus.11623

**Published:** 2020-11-22

**Authors:** Zachary Kirkland, Ranese E Jeffery, Jorge Conte, Justin George, Roberto Mercado

**Affiliations:** 1 Internal Medicine, Florida State University College of Medicine Internal Medicine Residency Program, Sarasota Memorial Hospital, Sarasota, USA; 2 Internal Medicine, Florida State University College of Medicine Internal Medicine Residency Program, Sarasota, USA; 3 Internal Medicine-Infectious Disease, Sarasota Memorial Healthcare System, Sarasota, USA

**Keywords:** herpes simplex, aseptic meningitis, viral meningitis, mollaret, meningitis monitoring, clinical characteristics

## Abstract

Aseptic meningitis is most commonly caused by herpes simplex virus (HSV), most often viral subtype 2. While typical meningeal symptoms include headache, photophobia/phonophobia, and nuchal rigidity, these are often much less severe than in bacterial meningitis. Rarely, patients may develop recurrent episodes of aseptic meningitis, sometimes with years between each presentation. A minimum of three episodes with at least one documented viral identification is classified as Mollaret meningitis. First described by Mollaret in 1945, the condition is self-limiting and often requires no intervention or suppressive antivirals. In fact, antiviral therapy may increase frequency of presentation. Our patient presented for her third bout of meningitis, with viral polymerase chain reaction positive for HSV-2 on lumbar puncture. The patient was successfully managed with supportive care without further suppressive antiviral therapy. As the disease is self-limiting, clinician education can mediate patient expectations, reduce unnecessary antiviral usage, and decrease superfluous healthcare resource utilization.

## Introduction

Aseptic meningitis is a clinical disease characterized by inflammation of the dura, the membranous covering of the spinal cord and cerebrospinal fluid (CSF), and negative bacterial cultures. Specific causes include fungi, malignancies, and, most often, viral infections. Mollaret meningitis is a rare, benign, recurrent, and self-limiting form of aseptic meningitis most often caused by herpes simplex virus type 2 [1]. Disease course varies wildly in reported cases, with some patients experiencing only three episodes and others reporting over 20 episodes. Diagnosis is established through clinical history and PCR confirmation of viral infection. Fortunately, symptoms tend to be mild, with most patients noting typical headache, meningismus, and photosensitivity. We present a case of a young female experiencing her third episode of aseptic meningitis. By raising awareness of this disease, practitioners and patients themselves should be able to assess current symptoms and if these symptoms require hospitalization or antibiotics. In doing so, unnecessary utilization of antibiotics and healthcare services can be minimized through patient education and reassurance. Here we discuss both the inpatient and outpatient management of this disease.

## Case presentation

A 30-year-old female presented to the ED for one day of neck pain, stiffness, headache, and malaise. She reported her headache as diffuse and pressure-like in nature. She denied any fever, chills, rigors, recent upper respiratory infection (URI), changes to vision, or syncope. Her past medical history was significant for two prior episodes of aseptic meningitis in 2013 and 2017, respectively. During her second hospitalization from meningitis, in 2017, she was diagnosed with HSV-2 confirmed on CSF PCR. At that time, she was treated with IV acyclovir 650 mg every eight hours for 10 days, achieving total resolution of symptoms. She denied any alleviating factors to her current symptoms, but stated her pain was provoked by light and sound. She also noted neck pain/stiffness aggravated by neck motion or bending forward. She rated her pain at 7/10. She felt her symptoms were very similar to her last episode of meningitis, which prompted the decision to seek care. Her medications, family history, and social history were non-contributory.

On arrival, her temperature was 37.3°C, blood pressure was 116/70 mmHg, heart rate of 80/min, respiratory rate of 16/min, and pulse oxygen saturation of 100% on room air. Pertinent physical exam findings include 2+ upper and lower reflexes, 5/5 muscle strength bilaterally, intact sensation, and pain with neck flexion. Kernig’s sign and Brudzinski sign were mildly positive. Fundoscopic exam revealed no papilledema but did elicit moderate discomfort.

**Figure 1:**
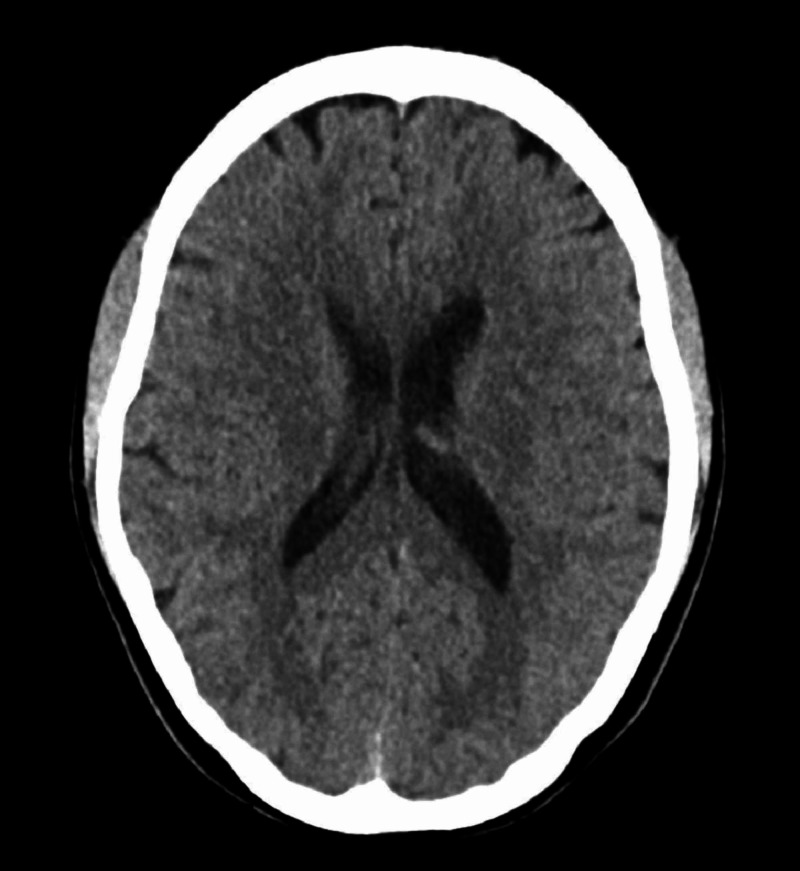
CT head w/o contrast demonstrating no acute findings

Upon initial presentation, labs revealed leukocytosis (7.2 K/uL), with differential included 68% segmented neutrophils, 20% lymphocytes, 10% monocytes, 1% eosinophils, 1% basophils. She was also found to have a mild normocytic anemia (Hgb 11.1K/uL, hematocrit 33.4%) that was later contributed to iron deficiency anemia associated with heavy menstruation. CT head without contrast showed no evidence of acute process, signs suggestive of infection, or obvious masses. In the emergency department, bedside lumbar puncture yielded a CSF analysis which showed 283 WBCs (predominately lymphocytes per pathologist review), 100 monocytes, elevated protein of 66g/dL, and normal glucose of 48mg/dL. Meningitis/encephalitis panel was positive for HSV-2, confirmed with CSF PCR once more. After discussed with the patient regarding the controversial use of antiviral agents, IV acyclovir 650 mg every eight hours was instituted by infectious disease (Figure 1).

Because of her previous history and LP cytology, empiric antibiotics were not started on admission. After admission, the CSF cultures grew micrococcus spp. and Acinetobacter Iwoffii. Prior to speciation, the patient was treated empirically with IV vancomycin 1250 mg every 12 hours and ceftriaxone 2 g every 12 hours. Subsequently, the bacteria were attributed to contamination and antibiotics were discontinued. After completing seven days of IV acyclovir, the patient’s symptoms improved and she was discharged home without suppressive antivirals.

## Discussion

Aseptic meningitis can be caused by fungi, mycobacterium, spirocetes, malignancy, and most commonly by viruses. In particular, enteroviruses (EV) and herpesviridae are the most common viral agents causing the disease [2]. Symptoms typically present as a much milder form of bacterial meningitis. While many viruses can cause aseptic meningitis, recurrent episodes are termed “Mollaret syndrome” or “Mollaret meningitis” [3]. Most often, this form of aseptic meningitis is secondary to HSV-2, confirmed with PCR of CSF. Mollaret meningitis is suggested to occur in approximately 20% of those with primary HSV-2 infections with meningismus [3]. This clinical presentation was first identified in 1944 by Mollaret, a French physician, with typical characteristics including diffuse headache, fever, and meningismus. In addition, current diagnostic criteria have been further expanded to include: recurrent attacks separated by symptom-free weeks or months and spontaneous remission of symptoms and signs [4]. Likewise, episodes are isolated and are usually separated by several weeks to months or even years. Approximately 85% of patients with primary HSV-2 meningitis experience active herpetic lesions in the days prior to meningismus. Despite this high percentage, herpetic lesions are not required for diagnosis of Mollaret meningitis or aseptic meningitis in general [5]. Our patient did report a non-vesicular rash on her lower back approximately one week prior to onset of symptoms but unfortunately it had resolved prior to her presentation so it could not be investigated.

A member of the herpesviridae, HSV-2 is an enveloped, double-stranded DNA virus classically associated with genital sores, although oral ulcers are becoming increasingly more common. Like most members of the herpesviridae, HSV-2 infections are lifelong, with viruses lying dormant within neural ganglia (e.g., dorsal root ganglia, trigeminal ganglia, etc.). By expressing latency-associated transcript (LAT) RNA, the programmed cell death mechanisms are downregulated and the virus is able to survive for many years within host cells without replication. While the current causes of viral reactivation are relatively unknown, periods of stress tend to cause reactivation of these previously quiescent infections.

Other viruses in the herpesviridae such as HHV-6, varicella zoster, and cytomegalovirus have been hypothesized as potential causes of recurrent aseptic meningitis because they can be lysogenic within the central nervous system for many years [6]. However, a literature review performed by the authors of this case report failed to yield any further case reports with identified agents other than HSV-1 or HSV-2. 

Like any meningeal infection, extreme caution must be used until CSF analysis rules out bacterial etiologies. Thus, most patients are treated empirically with empiric antibiotics. After a diagnosis of aseptic meningitis is made and the patient fulfills the diagnostic criteria of Mollaret's meningitis, treatment is primarily supportive, with emphasis on patient education and management of expectations. The use of antiviral therapies in these patients may actually increase the frequency of meningeal episodes [7]. However, for patients with compromised immunity, there is some data showing possible benefit, while administration of antivirals to immunocompetent patients has not shown any clear benefit [8]. Therefore, it is imperative that clinicians judiciously use these therapies and counsel their patients on the risks and benefits related to them.

## Conclusions

Because of its self-limiting nature, there are no guideline-directed therapy regimens for Mollaret meningitis. Prior to meeting diagnostic criteria (i.e., the first or second episode), treatment with IV acyclovir at 10 mg/kg every eight hours is often initiated. While some cases have shown a shortened disease course with IV antivirals, no data exists to definitively support their use in recurrent disease. While the role of antiviral therapy is supported in immunocompromised individuals due to reduced risk of neurologic sequelae, it is not supported in immunocompetent individuals. As with our case, initial treatment was given after a discussion with the patient given her persistent symptoms. The role of suppressive therapy with agents such as acyclovir or valacyclovir is controversial, especially because symptoms are typically self-limiting. Some studies support the use of suppressive therapy, although others suggest these agents may actually increase the frequency of meningitis. After another discussion with the patient about the risks and benefits of suppressive therapy, the decision was made to discharge her without oral antivirals. Additional therapies, such as estrogen, antihistamines, glucocorticoids, and colchicine have proven unsuccessful as both acute treatment and suppression, although again, the current data does not support their use in every patient. Interestingly, there are some reports of recurrent episodes after being asymptomatic for over 20 years. However, most patients experience the disease for three to five episodes, with no further episodes throughout their lives. Prognosis in Mollaret's meningitis is favorable, with most patients achieving remission of symptoms quickly with no lasting neurologic deficits.
